# The PREgnancy and FERtility (PREFER) study: an Italian multicenter prospective cohort study on fertility preservation and pregnancy issues in young breast cancer patients

**DOI:** 10.1186/s12885-017-3348-8

**Published:** 2017-05-19

**Authors:** Matteo Lambertini, Paola Anserini, Valeria Fontana, Francesca Poggio, Giuseppina Iacono, Annalisa Abate, Alessia Levaggi, Loredana Miglietta, Claudia Bighin, Sara Giraudi, Alessia D’Alonzo, Eva Blondeaux, Davide Buffi, Francesco Campone, Domenico F. Merlo, Lucia Del Mastro

**Affiliations:** 1 Department of Medical Oncology, U.O. Sviluppo Terapie Innovative, IRCCS AOU San Martino-IST, Largo Rosanna Benzi, 10, 16132 Genova, Italy; 2 Breast Cancer Translational Research Laboratory, Department of Medicine, Institut Jules Bordet, and l’Université Libre de Bruxelles (U.L.B.), Brussels, Belgium; 3 Physiopathology of Human Reproduction Unit, IRCCS AOU San Martino-IST, Genova, Italy; 4 Department of Epidemiology, Biostatistics and Clinical Trials - IRCCS AOU San Martino-IST Istituto Nazionale per la Ricerca sul Cancro, Genova, Italy; 5 Department of Medical Oncology, U.O. Oncologia Medica 2, IRCCS AOU San Martino-IST, Genova, Italy; 6 Department of “Alta intensità di Cura e Percorso Nascita”, U.O.C. Ostetricia e Ginecologia, UOSD Centro di Medicina Fetale e Perinatale, IRCCS Istituto Giannina Gaslini, Genova, Italy; 7 Department of “Alta intensità di Cura e Percorso Nascita”, U.O.C. Patologia e Terapia Intensiva Neonatale-Assistenza Neonatale, IRCCS Istituto Giannina Gaslini, Genova, Italy

**Keywords:** Breast cancer, Young patients, Fertility preservation, Pregnancy, Pregnancy-associated breast cancer

## Abstract

**Background:**

Fertility and pregnancy issues are of key importance for young breast cancer patients. Despite several advances in the field, there are still multiple unmet needs and barriers in discussing and dealing with these concerns. To address the significant challenges related to fertility and pregnancy issues, the PREgnancy and FERtility (PREFER) study was developed as a national comprehensive program aiming to optimize care and improve knowledge around these topics.

**Methods:**

The PREFER study is a prospective cohort study conducted across several Italian institution affiliated with the Gruppo Italiano Mammella (GIM) group evaluating patterns of care and clinical outcomes of young breast cancer patients dealing with fertility and pregnancy issues. It is composed of two distinctive studies: PREFER-FERTILITY and PREFER-PREGNANCY. The PREFER-FERTILITY study is enrolling premenopausal patients aged 18–45 years, diagnosed with non-metastatic breast cancer, who are candidates to (neo)adjuvant chemotherapy and not previously exposed to anticancer therapies. The primary objective is to obtain and centralize data about patients’ preferences and choices towards the available fertility preserving procedures. The success and safety of these strategies and the hormonal changes during chemotherapy and study follow-up are secondary objectives. The PREFER-PREGNANCY study is enrolling survivors achieving a pregnancy after prior history of breast cancer and patients diagnosed with pregnancy-associated breast cancer (PABC). The primary objectives are to obtain and centralize data about the management and clinical outcomes of these women. Patients’ survival outcomes, and the fetal, obstetrical and paediatric care of their children are secondary objectives. For both studies, the initial planned recruitment period is 5 years and patients will remain in active follow-up for up to 15 years. The PREFER-FERTILITY study was first activated in November 2012, and the PREFER-PREGNANCY study in May 2013.

**Discussion:**

The PREFER study is expected to support and improve oncofertility counseling in Italy, to explore the real need of fertility preserving procedures, and to acquire prospectively more robust data on the efficacy and safety of the available strategies for fertility preservation, on the management of breast cancer survivors achieving a pregnancy and of women with PABC (including the possible short- and long-term complications in their children).

**Trial registration number:**

ClinicalTrials.gov identifier: NCT02895165 (Retrospectively registered in August 2016).

## Background

In women of reproductive age, breast cancer is the most common malignancy with approximately 11% of new cases diagnosed every year in patients with 45 years of age or younger [1]. The burden of breast cancer diagnosed at young age is compounded by fertility and pregnancy issues that may contribute to the great psychosocial distress seen in these patients [2]. To intervene in a timely manner for addressing these concerns is crucial to not negatively affect short- and long-term quality of life of young survivors and also their adherence to treatment and subsequent disease outcomes [3].

Young breast cancer patients have an increased risk of developing biologically aggressive subtypes of tumors [4, 5]. Consequently, they often need and receive multimodality treatments that can be associated with significant side effects such as a transient or permanent impairment of gonadal function and subsequent infertility [6]. At the time of breast cancer diagnosis, approximately 50% of patients are concerned about the possible risk of treatment-related premature ovarian failure and infertility and are interested in maintaining fertility and future reproductive capacity [7]. International guidelines recommend an early and prompt discussion about the possible risk of developing these side effects with all young patients who are candidates to receive anticancer therapies to help them with informed decisions on the available strategies for fertility preservation [8–10]. Embryo/oocyte cryopreservation, cryopreservation of ovarian tissue and the use of temporary ovarian suppression with luteinizing hormone-releasing hormone analogs (LHRHa) during chemotherapy are the available options to preserve fertility in breast cancer patients [11]. Despite a growing literature on this topic over the past years, there are still several obstacles limiting the access to fertility preservation procedures [11, 12]. Moreover, very limited data are available on the number of patients that take active steps towards the available strategies for fertility preservation and on the reasons for refusal of these procedures after oncofertility counseling. This still lacking information is crucial also from a public health perspective to better organize the network between oncology and fertility units and for resource allocation. Finally, of note, the currently available data on the safety and efficacy of fertility preserving strategies in cancer patients derive mainly from small single center retrospective or prospective series.

At the time of cancer diagnosis, approximately 50% of young breast cancer patients are willing to become pregnant after completing therapy [13]. However, breast cancer patients have the lowest chance of having a subsequent pregnancy among female cancer survivors, which is approximately 67% lower than the general population after adjusting for women’s age, education level and previous parity [14]. The frequent need for gonadotoxic chemotherapy and prolonged treatment periods up to 10 years after diagnosis with adjuvant endocrine therapy in women with hormone receptor-positive breast cancer are possible explanations for these findings. Moreover, among physicians, there is still a general misconception on the possible negative prognostic effect of pregnancy in patients with breast cancer being a hormonally driven disease, and on the possible negative impact of prior anticancer treatments on pregancy outcomes [12]. The available data on the topic do not support this concern and pregnancy after breast cancer should not be in principle discouraged but should be monitored closely [9, 15]. However, this recommendation is based mainly on retrospective data with no prospective studies conducted so far to investigate all the issues related to safety and monitoring of pregnancy in cancer survivors.

Pregnancy-associated breast cancer (PABC) is defined as breast cancer diagnosed during pregnancy or within 1 year after delivery. Breast cancer complicates between 1 in 3000 to 1 in 10,000 pregnancies and represents the most frequently diagnosed malignancy among pregnant women [16]. Population-based studies report an overall incidence of PABC ranging between 2.4 to 7.3 per 100,000 pregnancies [17–20]. Although being a relatively rare condition, the issue of PABC might become more common in the coming years due to evidence suggesting that the incidence of breast cancer in young women and the occurrence of PABC are increasing [21, 22]. Moreover, in western countries, there is a current trend of postponing pregnancy to later in life: a recent Italian study in a cohort of more than 2000 women showed that mean maternal age was 33 years with approximately 40% of pregnancies occurring after the age of 35 years [23]. The diagnosis of PABC represents a unique challenge for the patient, her caregivers and the treating physicians raising also several moral, social or religious issues that should be considered in the management of this complex condition [24]. In the last decade, important advances in the field of managing PABC have been made thanks to the effort of several groups that looked mainly into the safety of administering chemotherapy during pregnancy [25–27]. These important contributions in the field allowed the development of specific guidelines to help physicians in dealing with PABC [9, 22]. However, due to its relative rarity, several aspect of the clinical management of these patients are based on limited evidence and further research efforts are warranted.

Although a growing attention has been given to fertility and pregnancy issues in young breast cancer patients over the past years, several grey zones remain in many domains of this field and some physicians are still uncomfortable in dealing with these subjects. To address the significant challenges related to fertility and pregnancy issues, we have developed the PREgnancy and FERtility (PREFER) study, a comprehensive program aiming to optimize care and improve knowledge around these topics across Italy. The program was initiated at the IRCCS AOU San Martino-IST in Genova (Italy) and then it has been spread to other Italian Institutions under the umbrella of the Gruppo Italiano Mammella (GIM) group. This article aims to describe the study design and methodology, and the program that is being implemented in Italy on these topics according to available national guidelines.

## Methods/design

The PREFER study is a prospective cohort study conducted across several Italian institution affiliated with the GIM group aiming to optimize care and improve knowledge on fertility preservation and the management of pregnancy by evaluating the pattern of care and clinical outcomes of young breast cancer patients dealing with these issues. It is composed of two distinctive studies, one assessing fertility (i.e. PREFER-FERTILITY) and the other pregnancy (i.e. PREFER-PREGNANCY) issues. Hence, two different study protocols were developed under the umbrella of the PREFER study.

### PREFER - FERTILITY STUDY

#### Study design and setting

The PREFER-FERTILITY study  is a prospective cohort study designed to obtain and centralize data about the preferences and choices of young cancer patients on the fertility preservation strategies available in Italy as well as to assess the outcomes of women undergoing one or more of these options in terms of both success and safety of the techniques.

**Fig. 1:**
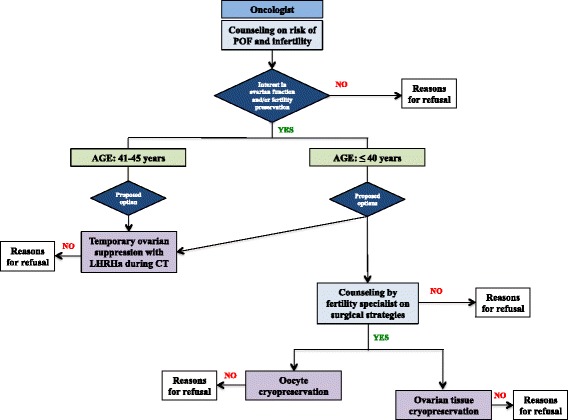
Suggested algorithm for physicians dealing with fertility issues. *POF* premature ovarian failure; *LHRHa* luteinizing hormone-releasing hormone analogs; *CT* chemotherapy

According to national guidelines for fertility preservation in cancer patients developed by the Italian Association of Medical Oncology (AIOM), the PREFER-FERTILITY study provides a specific suggested algorithm for physicians to deal with these issues (Fig. 1). As early as possible before the initiation of systemic treatments, the oncologists make young women with recently diagnosed breast cancer aware of the potential negative impact of anticancer therapies on ovarian function and fertility and evaluate their interests in ovarian function and/or fertility preservation. Due to the low success rate of cryopreserving procedures in women older than 40 years, only temporary ovarian suppression with LHRHa during chemotherapy is proposed to patients aged between 41 and 45 years who are concerned about the risk of developing treatment-related premature ovarian failure. In patients diagnosed at 40 years of age and younger, both the use of temporary ovarian suppression with LHRHa during chemotherapy and a complete reproductive counseling to access the cryopreserving procedures are offered. The choice between these two possibilities is not mutually exclusive, since more than one technique can be applied in the same patient. Patients who are potentially interested in the cryopreserving options are then referred to reproductive units for further complete counseling on the possibility to undergo oocytes cryopreservation or cryopreservation of ovarian tissue (i.e. in Italy, embryo cryopreservation is prohibited by law). Type of procedure, timing, possible complications, and expected results for each of the strategy is described to clarify to the patient what is known or still experimental about these techniques. A well-organized linkage between oncology and reproductive units is crucial to face the management of fertility issues in these patients. The implementation of this program is the first step to be accomplished for all the centers participating in the PREFER study.

#### Eligibility criteria

The PREFER-FERTILITY study is enrolling premenopausal patients diagnosed with breast cancer at a young age (defined as age between 18 and 45 years). Eligible patients should not present with metastatic disease and should not have received chemotherapy and/or radiation therapy prior to study initiation. Inability to provide written informed consent, diagnosis of de novo metastatic disease and the existence of severe psychiatric disorders are exclusion criteria. The eligibility criteria are intentionally broad for trying to exclude as few patients as possible so that true population-based data can be obtained.

#### Study objectives

The primary objective of the PREFER-FERTILITY study is to obtain and centralize data about the preferences and choices of young breast cancer patients on the fertility preservation strategies available in Italy and proposed by the oncologists. Information on reasons for refusal will be collected to gain a better understanding of factors that influence patients’ choice towards the available strategies for fertility preservation.

Secondary objectives of the PREFER-FERTILITY study are:

1) To evaluate the success of the available strategies for fertility preservation in terms of ovarian function recovery, number and quality of oocytes collected and cryopreserved, post-treatment pregnancies.

2) To evaluate the safety of the available strategies for fertility preservation in terms of disease-free survival (DFS) and overall survival (OS).

3) To determine the hormonal changes during chemotherapy and study follow-up through the evaluation of anti-Mullerian hormone (AMH), follicle-stimulating hormone (FSH), and estadiol (E2) at pre-specified timepoints (i.e. before chemotherapy initiation, after the first and second cycle of chemotherapy, at the end of chemotherapy and every 6 months during study follow-up).

#### Baseline evaluation and follow-up

Baseline patient demographic and tumor characteristics are collected at enrollment. Particular attention is given to the following information: menstrual history, presence of any pre-existing gynecological disease and treatment received, parity status, prior hormonal treatments or prior use of assisted reproductive technology (ART) for infertility. Subsequently, data on types of fertility preserving procedures offered at the time of cancer diagnosis, types of those accepted and refused by patients including reasons for refusal are collected. For patients undergoing oocyte cryopreservation procedures, information on the protocol used for controlled ovarian stimulation, patients’ response to treatment and the success of the procedures in terms of quality and quantity of oocytes collected and cryopreserved are retrieved. Finally, the study collects data on anticancer therapies received, hormonal changes, menstrual function and pregnancies during treatment and study follow-up, disease-status, and date of last follow-up or death.

An ad hoc electronic platform for centralized data collection has been created at the Clinical Trial Unit of the IRCCS AOU San Martino-IST in Genova (Italy). Specific electronic case report forms (e-CRF) for the PREFER-FERTILITY study are used to collect data. A password-protected system is used to provide the investigators with the access to the e-CRF.

#### Ethical considerations and progress of the study

The Ethics Committee of the coordinating center approved the PREFER-FERTILITY study protocol on November 23, 2012 (reference number: 001377). Then, ethical approval has been obtained from all participating institutions affiliated with the GIM group before study initiation in each center (Table 1). All patients must provide a written informed consent before study inclusion. The contract research organization responsible for the administrative aspects of all the GIM studies (i.e. Clinical Research Technology) manages also the PREFER-FERTILITY study.

**Table 1:** Name of the institutions participating in the PREFER-FERTILITY study

Name of the institution	City, country
IRCCS A.O.U. San Martino-IST	Genova, Italy
Ospedale Vito Fazzi	Lecce, Italy
ASL 1 Sassari	Sassari, Italy
A.O.U. Santa Maria della Misericordia	Udine, Italy
A.O. Carlo Poma	Mantova, Italy
A.O.S. Croce e Carle	Cuneo, Italy
A.O. Arcispedale Santa Maria Nuova - IRCCS	Reggio Emilia, Italy
Ospedale Versilia	Lido di Camaiore (Lucca), Italy
A.O.U. di Ferrara	Ferrara, Italy
ASS1 Triestina	Trieste, Italy
Istituto Nazionale Tumori - IRCCS Fondazione G. Pascale	Napoli, Italy
Ospedale Cardinal Massaia	Asti, Italy
A.O.U. Pisana	Pisa, Italy
Istituto di Candiolo – IRCCS, Fondazione del Piemonte per l’Oncologia	Candiolo (Torino), Italy
Presidio Ospedaliero Antonio Perrino	Brindisi, Italy
A.O.U. Federico II	Napoli, Italy
A.O. San Carlo	Potenza, Italy
Ospedale Sacro Cuore Don Calabria	Negrar (Verona), Italy
Ospedale Santa Maria della Misericordia	Perugia, Italy
IRCCS Fondazione Salvatore Maugeri	Pavia, Italy

The PREFER-FERTILITY study was first activated at the coordinating center in November 2012. To allow an adequate time to assess the feasibility of the project, the opening of the other centers started approximately 2 years after study initiation (March 2015). The initial planned recruitment period is 5 years and patients will remain in active follow-up for up to 15 years. A protocol amendment to prolong study recruitment period is currently being prepared. To reduce selection bias, all the centers that are enrolling patients are strongly adviced to systematically invite all eligible women to participate in the study.

#### Statistical analysis

The statistical analysis for the PREFER-FERTILITY study is mainly descriptive. Continuous variables will be summarized using summary statistics (i.e. mean, median and standard deviation) and, to test differences between groups when applicable, parametric t-test or F-test or nonparametric Kruskal-Wallis test or Wilcoxon’s rank sum test will be used. The Kaplan-Meier method will be used to estimate cumulative survival probabilities and generate survival curves; the log-rank test will be used to test for significance univariate analysis of differences between survival rates. The Cox proportional hazards model will be used to perform multivariate analysis for survival adjusting for potential confounders. Parameter estimates will be reported together with 95% confidence intervals. All tests will be two-sided and a *p* value of <0.05 will be considered statistically significant.

### PREFER - PREGNANCY STUDY

#### Study design and setting

The PREFER-PREGNANCY study  is a prospective cohort study designed to obtain and centralize data on two major issues: 1) the clinical outcomes of breast cancer survivors that achieve a pregnancy after prior diagnosis and treatment of breast cancer including the outcomes of their pregnancies; 2) the management of PABC, including fetal, obstetrical and paediatric care of children born after prior in utero exposure to anticancer treatments, and the long-term survival outcomes of these patients.

For breast cancer survivors achieving a pregnancy at the completion of anticancer treatments, no specific AIOM guidelines have been developed. The only recommendation is that, once pregnancy has occurred, induction of abortion has no therapeutic role and should be strongly discouraged. Moreover, in patients with endocrine sensitive disease, the interruption of endocrine therapy outside a clinical trial is contraindicated.

For the management of PABC, physicians are encouraged to follow the national AIOM guidelines on the topic developed from the international guidelines [9, 22]. While the management of women diagnosed within 1 year after delivery should not differ from that of premenopausal patients diagnosed outside pregancy (with the only exception that breastfeeding is contraindicated while receiving anticancer treatments), specific recommendations should be followed for women diagnosed with breast cancer during pregnancy.

**Fig. 2:**
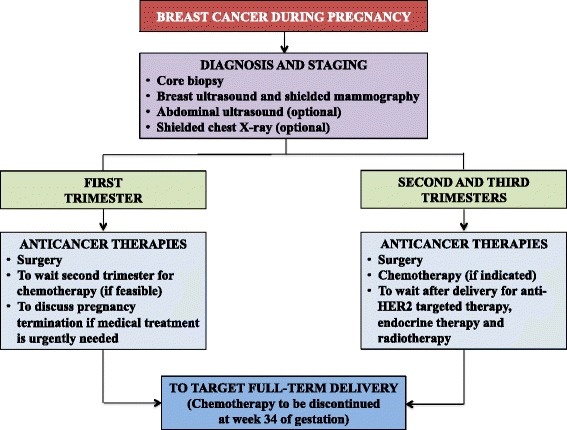
Suggested algorithm for the management of patients with breast cancer diagnosed during pregnancy

In women with breast cancer diagnosed while pregnant (Fig. 2), histopathologic diagnosis based on core biopsy is the gold standard and should follow standard procedures as in non-pregnant patients, but informing the pathologist about the pregnancy status. As imaging procedures for diagnosis, breast ultrasound and mammography with abdominal shielding are allowed while contrast-enhanced breast magnetic resonance imaging (MRI) is contraindicated in this setting. Ultrasound is also the preferred imaging modality for staging abdomen and pelvis, with the possibility to perform also chest X-ray with abdominal shielding. Computed tomography, bone scan and positron emission tomography should be avoided in women with breast cancer diagnosed while pregnant.

Regarding anticancer treatments, surgery can be safely performed at any time during the course of gestation and should follow the same guidelines as for non-pregnant cases. Adjuvant loco-regional radiotherapy should be post-poned to the postpartum period. Among the systemic treatments, chemotherapy (i.e. anthracycline-based or anthracycline/taxane-based regimens) is contraindicated in the first trimester, but it can be safely administered during the second and third trimesters. For patients diagnosed in the first trimester with urgent need to start systemic therapy, therapeutic abortion needs to be discussed. To avoid delivery during the nadir period, a 3-week interval between the last dose of chemotherapy and the expected date of delivery should be allowed: hence, chemotherapy should be discontinued at week 34 of gestation. The goal is to achieve a full-term delivery (i.e. after week 37 of gestation). Elective administration of anti-HER2 targeted therapy as well as endocrine therapy should be avoided during pregnancy and should be postponed after delivery.

Since systemic cytotoxic therapy can be associated with an increased risk of obstetric and fetal complications, pregnancy in cancer patients should be considered and monitored as “high risk” with a fetal anomaly and growth scan (i.e. ultrasound) at least every 3–4 weeks to monitor fetal well-being, growth and general development. Fetal MRI in the presence of abnormalities and cardiotocography in the case of intrauterine growth retardation should be considered. Although mode of delivery should not differ from usual obstetric indications, delivery in a tertiary center is the suggested option; histological evaluation of the placenta is recommended to assess possible breast cancer cell contamination. Finally, a correct monitoring for the possible occurrence of short- and long-term complications in children with in utero exposure to anticancer treatments is strongly suggested.

#### Eligibility criteria

The PREFER-PREGNANCY study is enrolling patients diagnosed with breast cancer during pregnancy or within 1 year after delivery (i.e. PABC) or survivors who achieve a pregnancy after prior diagnosis and treatment for breast cancer. In the PREFER-PREGNANCY study, patients presenting with de novo metastatic disease are not excluded. The only exclusion criteria are any inability to provide written informed consent and the existance of severe psychiatric disorders.

#### Study objectives

The primary objectives of the PREFER-PREGNANCY study are to obtain and centralize data about the management and clinical outcomes of both breast cancer survivors that achieve a pregnancy after prior diagnosis and treatment for breast cancer and PABC.

Secondary objectives are to evaluate the fetal, obstetrical and paediatric care of children born in breast cancer survivors and of those previously exposed in utero to anticancer treatments.

#### Baseline evaluation and follow-up

The PREFER-PREGNANCY study collects information on patient demographic and tumor characteristics, as well as cancer-related treatment data. For patients diagnosed with breast cancer during pregnancy, more detailed information on the anticancer treatments administered during pregnancy are collected. Particular attention is given to the following information: type of conception, type of tests performed during pregnancy, pregnancy outcomes with gestational date at delivery, and pregnancy or obstetrical complications. Subsequently, data on the health newborn, as well as the growth and development of these children are collected during pediatric follow-up. Information on fetal, obstetrical and paediatric care of children born from these patients is retrieved. Finally, data on further anticancer treatment received, menstrual function and further pregnancies during treatment and study follow-up, disease status, and date of last follow-up or death are collected.

The same electronic platform with related access through a password-protected system as for the PREFER-FERTILITY study is used for centralized data collection also in the PREFER-PREGNANCY study at the Clinical Trial Unit of the IRCCS AOU San Martino-IST in Genova (Italy). Two separate e-CRF can be accessed for collecting data within the PREFER-PREGNANCY study: one is dedicated to patients achieving pregnancy after prior diagnosis and treatment of breast cancer and the other for women diagnosed with PABC.

#### Ethical considerations and progress of the study

The Ethics Committee of the coordinating center approved the PREFER-PREGNANCY study protocol on May 28, 2013 (reference number: 000650). Then, ethical approval has been obtained from all participating institutions affiliated with the GIM group before study initiation in each center (Table 2). All patients must provide a written informed consent before study inclusion. Clinical Research Technology manages also the PREFER-PREGNANCY study.

**Table 2:** Name of the institutions participating in the PREFER-PREGNANCY study

Name of the institution	City, country
IRCCS A.O.U. San Martino-IST	Genova, Italy
Ospedale Vito Fazzi	Lecce, Italy
ASL 1 Sassari	Sassari, Italy
Fondazione Poliambulanza	Brescia, Italy
A.O.U. Santa Maria della Misericordia	Udine, Italy
A.O. Carlo Poma	Mantova, Italy
A.O.S. Croce e Carle	Cuneo, Italy
A.O. Arcispedale Santa Maria Nuova - IRCCS	Reggio Emilia, Italy
Ospedale Versilia	Lido di Camaiore (Lucca), Italy
A.O.U. di Ferrara	Ferrara, Italy
ASS1 Triestina	Trieste, Italy
A.O.U. Pisana	Pisa, Italy
Istituto di Candiolo – IRCCS, Fondazione del Piemonte per l’Oncologia	Candiolo (Torino), Italy
Presidio Ospedaliero Antonio Perrino	Brindisi, Italy
A.O.U. Federico II	Napoli, Italy
A.O. San Carlo	Potenza, Italy
Ospedale Sacro Cuore Don Calabria	Negrar (Verona), Italy
IRCCS Fondazione Salvatore Maugeri	Pavia, Italy
Istituto Nazionale Tumori Regina Elena - IRCCS	Roma, Italy

The PREFER-PREGNANCY study was first activated at the coordinating center in May 2013. As for the PREFER-FERTILITY study, to allow an adequate time to assess the feasibility of the project, the opening of the other centers started approximately 2 years after study initiation (March 2015). The initial planned recruitment period is 5 years and patients will remain in active follow-up for up to 15 years. A protocol amendment to prolong study recruitment period is currently being prepared. To reduce selection bias, all the centers that are enrolling patients are strongly adviced to systematically invite all eligible women to participate in the study.

#### Statistical analysis

Similar consideration as for the PREFER-FERTILITY study can be done. Within the PREFER-PREGNANCY study, the two scenarios of pregnancy in breast cancer survivors and PABC will be considered and analyzed separately.

## Discussion

Concerns regarding fertility and pregnancy are key issues in young breast cancer patients and are now becoming increasingly important. Several advances in these fields have been made over the past years. However, there are still several unmet needs and barriers remain in discussing and dealing with these issues. The PREFER study represents a comprehensive program in young breast cancer patients across several Italian institutions aiming to optimize care and improve knowledge in the fields of fertility preservation, management of pregnancy in breast cancer survivors and PABC.

Professional guidelines recommend that all young patients should be advised on the fertility threat of their cancer care [8–10]. Several services and resources are available to help oncologists in addressing these issues with patients and to improve adherence to guidelines [28–32]. Nevertheless, oncologists face several barriers to have this discussion, including lack of knowledge and safety concerns, insufficient resources and lack of linkage with reproductive units [33]. As recently shown in an Italian survey, 93% of medical oncologists acknowledged having poor insight into the subject, more than 80% were not in favor of performing a hormonal manipulation for cryopreservation procedures, and 90% underscored a lack of coordination between oncology and reproductive units [12]. The PREFER-FERTILITY study aims to support and to improve the discussion around fertility issues among oncologists and patients before treatment with the ultimate goal to implement the referral of young women interested in fertility preserving procedures to reproductive unit. As recently shown in the United States of America, the development of a fertility program to support clinicians in discussing fertility issues improved patient satisfaction with information received and the quality of oncofertility counseling [34].

To date, despite the recognition of the importance of fertility preservation in young cancer patients, limited information exists on the actual number of patients that, following oncofertility counseling, accept to undergo one of the available strategies for fertility preservation and the reasons for refusal (i.e. primary objective of the PREFER-FERTILITY study). To achieve more information on this regard would be fundamental for improving the quality of oncofertility counseling [35]. Moreover, these findings would serve as crucial information from a public health perspective for a better resource allocation giving a point estimate on the workload needed on this regard. A well-organized network between oncology and reproductive units is fundamental [15]; however, it remains unknown if this should be implemented on a local basis or should be centralized on a regional/national level.

Regarding the efficacy and safety of the available strategies for fertility preservations (i.e. secondary objectives of the PREFER-FERTILITY study), limited and mainly retrospective data exist in the oncologic population. Only one prospective study investigated the efficacy and safety of performing a controlled ovarian stimulation for embryo cryopreservation in breast cancer patients [36, 37]. The study showed that pregnancy rates with the use of embryo cryopreservation in breast cancer patients are comparable to those expected in a non oncologic population [36]. Moreover, no negative impact on patients’ survival was observed with the use of a controlled ovarian stimulation before the initiation of anticancer treatments [37]. However, the numbers remain low to draw solid conclusions and even more limited data exist for oocyte cryopreservation, the only standard cryopreservation strategy that can be applied in Italy [38]. Similarly, there is lack of data﻿ on cryopreservation of ovarian tissue [39]. This is the only available option for fertility preservation in prepubertal girls who are candidates to gonadotoxic anticancer treatments [40]. The technique is considered experimental in adult cancer patients [8, 9], but might be proposed to selected women such as those who cannot delay anticancer treatments or with contraindications to controlled ovarian stimulation [15]. Nevertheless, limited data exist on its efficacy and safety in the specific subgroup of breast cancer patients. Finally, the efficacy of temporary ovarian suppression with LHRHa during chemotherapy in breast cancer patients has been recently supported by two randomized studies and a large meta-analysis [41–43]. In Italy, the AIOM society recommends its use and the 6-month treatment during chemotherapy is covered by the National Health Care System [44]. Temporary ovarian suppression with LHRHa during chemotherapy is the most used fertility preserving technique by Italian oncologists: a total of 86% of the surveyed physicians favored its use and 65% declared to use it regularly [12]. However, long-term fertility and survival outcomes with the use of this strategy are still limited and a prospective collection of these outcomes would give further insights on the efficacy and safety of the procedure.

According to experts’ recommendations, pregnancy after prior diagnosis and treatment for breast cancer should not be in principle discouraged but should be monitored closely [9, 15]. Nevertheless, despite an increased awareness on its feasibility, the number of breast cancer survivors achieving a subsequent pregnancy remains low. Several barriers remain in this field beyond the impact of anticancer treatments on fertility potential. Only 54% of the surveyed Italian oncologists believed that pregnancy does not affect the prognosis of breast cancer survivors and 40% agreed with the statement that a higher percentage of fetal malformation and pregnancy complications can be present in pregnancies occurring in breast cancer survivors [12]. However, the retrospective evidence available on this issue suggests that pregnancy in cancer survivors is safe, also in women with hormone receptor-positive disease [45]. Moreover, the neonatal outcomes in cancer survivors seem not to differ from those of the general population; nevertheless, a relatively higher abortion rate and incidence of birth complications were observed in this population as compared to untreated women [46, 47]. Of note, the lack of prospective data on this topic remains an important concern that needs to be overcome. The PREFER-PREGNANCY study aims to prospectively acquire information on number of breast cancer survivors achieving pregnancy during oncologic follow-up, and to evaluate the clinical outcomes of these women and their pregnancies. Another important unanswered issue in this field, especially for women with hormone receptor-positive disease, is the ideal interval to wait between the end of anticancer treatments and the conception. An ongoing international prospective study conducted by the International Breast Cancer Study Group (IBCSG), with the collaboration of the Breast International Group (BIG) and the North American Breast Cancer Group (NABCG) is currently trying to answer this important question (the POSITIVE study) [48]. This study is dedicated to the specific subgroup of breast cancer patients with hormone receptor-positive disease; the main aim is to evaluate the feasibility and safety of a temporary interruption of endocrine therapy to allow pregnancy after 18 to 30 months of treatment [48]. The results of these prospective efforts are awaited to implement recommendations on the best management of these patients and the monitoring of their pregnancies.

PABC is a complex medical situation requiring the involvement of a multidisciplinary team with all different specialties since the early phases [9, 22]. A correct application of the available guidelines for the diagnosis, staging, and treatment of PABC is crucial to manage correctly this critical clinical situation [9, 22]. Despite the important advances made in the last years, current guidelines rely on limited evidence and several questions remain unanswered in this field. Prospective studies, like the one organized in Europe by the International Network on Cancer, Infertility and Pregnancy (https://www.esgo.org/network/incip/), are currently ongoing to investigate the management of PABC. The PREFER-PREGNANCY study represents another prospective effort on this regard with the aim to centralize data on the management of patients with PABC across several Italian centers. The impossibility of conducting randomized study in this setting highlights the importance to participate in these prospective registries that will give the opportunity to accrue adequate numbers for reaching more robust evidence on the management of women with PABC as well as on the the possible occurrence of short- and long-term complications in children with in utero exposure to anticancer treatments.

In conclusion, the PREFER study represents a comprehensive program dedicated to young breast cancer patients and conducted across several Italian institutions aiming to optimize care and improve knowledge in the field of fertility preservation, management of pregnancy in breast cancer survivors and PABC. The PREFER study provides a unique opportunity to support and improve oncofertility counseling in Italy and to explore the real need of fertility preserving procedures. Furthermore, the study gives the chance to acquire prospectively more robust data on the efficacy and safety of the available strategies for fertility preservation, on the management of breast cancer survivors achieving a pregnancy and of women with PABC including the possible occurrence of short- and long-term complications in children with in utero exposure to anticancer treatments.

## Abbreviations

AIOM: Italian Association of Medical Oncology
AMH: Anti-Mullerian hormone
ART: Assisted reproductive technology
BIG: Breast International Group
DFS: Disease-free survival
E2: Estadiol
e-CRF: Electronic case report forms
FSH: Follicle-stimulating hormone
GIM: Gruppo Italiano Mammella
IBCSG: International Breast Cancer Study Group
LHRHa: Luteinizing hormone-releasing hormone analogs
MRI: Magnetic resonance imaging
NABCG: North American Breast Cancer Group
OS: Overall survival
PABC: Pregnancy-associated breast cancer
PREFER: PREgnancy and FERtility
